# 81. Fabry disease presenting as autoimmune rheumatic disease

**DOI:** 10.1093/rap/rky034.044

**Published:** 2018-09-20

**Authors:** Jennifer Hannah, Shirish Sangle, David D'Cruz

**Affiliations:** Rheumatology, Guys and St Thomas NHS Trust, London, UNITED KINGDOM

**Introduction:** Fabry disease is an X-linked lysosomal storage disorder caused by deficiency of galactosidase A activity resulting in accumulation of globotriaosylceramide and related glycosphingloipids. Progressive accumulation of glycosphingolipids in the vascular endothelium results in ischaemia and infarction of small vessels due to vascular occlusion, with particularly damaging effect to the kidneys, heart and brain. Substantial deposition in podocytes leads to proteinuria, and in cardiomyocytes leads to cardiac hypertrophy and conduction abnormalities. Early clinical manifestations in childhood and adolescence include intermittent pain in the extremities (acroparesthesiae), angiokeratomas, corneal opacity, hypohidrosis, heat, cold and exercise intolerance, proteinuria, and gastrointestinal disturbance. By adulthood, patients may have developed end-stage renal failure and/or severe cardiac or cerebrovascular disease, although milder, atypical variants also exist that present later in life (>40 years) with cardiomegaly and mild proteinuria without any of the earlier symptoms. Males tend to have a more severe disease phenotype, although some heterozygotic females also exhibit severe disease due to random X-chromosomal inactivation. It is a rare condition with incidence estimates ranging from 1 in 40,000 to 60,000 males. Despite symptoms usually starting in childhood, they are often misattributed to other conditions, especially in the absence of a family history when the average age of diagnosis is 29 years. The Fabry Outcome Survey of all consenting patients receiving or eligible for enzyme replacement therapy in Europe found that 25% were originally misdiagnosed, and 39% of misdiagnoses were rheumatological diseases. We describe two patients who received delayed diagnosis of their Fabry’s disease but who appear to still have a genuine diagnosis of an autoimmune rheumatic disease alongside Fabry disease.

**Case description:** Patient A - Fabry disease with antiphospholipid syndrome

Patient A was a previously healthy 47 year old female patient when she presented to A&E on multiple occasions with recurrent episodes of acute chest pain with elevated troponin and ECGs showing static T wave inversion in the lateral leads. Repeated coronary artery angiograms and an intra-vascular ultra-sound failed to show any significant coronary artery narrowings. Echocardiogram, V/Q scan and CTPA were all normal. She had a persitent tropinaemia with a normal CK and LDH. Further history elicited a background of four pregnancies, including a miscarriage at 10 weeks and a significant post-partum haemorrhage with another. She had arthralgia with early morning stiffness lasting one to two hours, longstanding hair fall, and extreme fatigue. IgM anticardiolipin was positive and the lupus anticoagulant was strongly positive. Anti-nuclear antibody (ANA) was weakly positive in a nucleolar pattern (though subsequent tests have been negative). Crithidia dsDNA, Ro (SSA), La (SSB), RNP, Sm, Scl-70, Jo-1 and Ribosomal antibodies were not detected. Complement C3/C4 levels were normal. Cardiac MRI confirmed a mid-wall area of a late Gadolinium enhancement and a mild pericardial effusion - consistent with inflammatory perimyocarditis. The diagnosis was made of cardiac anti-phospholipid syndrome with inflammatory myocarditis. Despite mycophenolate and hydroxychloroquine immunosuppression, her symptoms persisted and a repeat cardiac MRI three years after starting mycophenolate, showed increased scar volume and evidence of ongoing inflammation. At this point a left ventricular biopsy was arranged which showed extensive myocyte vacuolisation, interstitial fibrosis and scar formation. These findings are non-specific but it was felt to be consistent with hydroxychloroquine-induced cardiomyopathy, and her troponin and BNP levels did show improvement upon stopping hydroxychloroquine. Two years later, she has been troubled by runs of non-sustained ventricular tachycardia. A repeat MRI demonstrated left ventricular hypertrophy. At this point alpha galactosidase testing was performed and she was found to have a heterozygotic C644A-6 mutation in GLA gene consistent with Fabry disease. Immunosuppression has been stopped and she has now been started on enzyme replacement therapy. Warfarin can also be stopped as it is not indicated in purely obstetric anti-phospholipid syndrome, however the patient has chosen to continue this.

Patient B - Fabry disease with SLE and lupus nephritis

Patient B is a 40 year old female. In 2001 she had a PE and DVT and a couple of years later developed a post-partum pericardial effusion with renal abnormalities identified at another hospital. She underwent a renal biopsy in 2003 which showed focal proliferative WHO class III lupus nephritis. The presence of Fabry-like cytoplasmic inclusions was also noted on the biopsy, although chloroquine toxicity was considered as a differential. Fabry disease was confirmed by a low alpha-galactosidase level and genetic testing revealed a heterozygote novel missense mutation in the GLA gene. She was diagnosed with SLE and managed with prednisolone, azathioprine and hydroxychloroquine. In 2010 she developed widespread lymphadenopathy, neutropenia, night sweats and fevers, and a fine needle aspirate of the lymph nodes demonstrated a reactive picture. Azathioprine was stopped due to the significant neutropenia. There was some initial improvement in neutrophil count following increasing her steroid dose suggesting an autoimmune aetiology of her neutropenia, although ANA was negative at this time. She intermittently suffers from arthralgias, sweats and fevers, weight loss and lymphadenopathy. Subsequent ANA has been positive (up to 1:320 dilution) and further immunological testing supports the diagnosis of SLE with Anti-Ro, Anti-C1q and dsDNA being present and C3 and C4 complement levels intermittently low. Lupus anticoagulant is positive but anticardiolipin antibodies are negative. In 2012 she developed chest pain on exertion. Resting ECG showed T wave inversion in lateral chest leads, I and aVL. A cardiac MRI showed extreme myocardial oedema and she underwent a left ventricular biopsy which did not demonstrate any inflammatory cell infiltration, complement or immunoglobulin deposition. In view of her SLE background she was managed as lupus myocarditis by increasing her prednisolone dose. Further immunosuppressants were not administered due to her persistently low neutrophil count (0.2) However, questions remain regarding whether the Fabry disease may be the underlying cause. A repeat cardiac MRI showed ongoing myocardial oedema with apical hypertrophic change with T2 enhancement suggesting Fabry disease. Ophthalmology review confirmed bilateral inferior episcleral microaneurysms consistent with Fabry disease. In 2013, she had a significant flare of her renal disease and repeat biopsy showed active Class III and Class V nephritis. Segmental sclerosing lesions were seen within the glomeruli which would be in keeping with either previous active lupus but also Anderson-Fabry disease given the presence of Fabry-type inclusions in swollen protocytes. On two separate occasions in 2017, she developed infected hair follicles alongside significant neutropenia. Following antibiotic therapy with GCSF she then developed a severe thrombocytopenia and which responded well to IVIg and she was started on mycophenolate mofetil (MMF). She currently remains stable on MMF from a renal, haematological and cardiac perspective and has not required any enzyme replacement therapy.

**Discussion:** Fabry disease is multi-systemic and shares common symptoms with autoimmune rheumatic diseases, for example fatigue (62%) and neuropathic pain (77%). The co-existence of Fabry disease and lupus nephritis histology on renal biopsy has previously been reported. It has also been shown that there is an increase in thrombotic tendency in Fabry patients (12%). Autoantibodies are commonly seen in Fabry disease. In a cohort of Argentinian Fabry disease patients, 57% patients showed reaction to at least one autoantibody (extractable nuclear antigen, double-stranded DNA, anticardiolipin or phosphatidylserine) with 45% patients having detectable anti-phospholipid antibodies. However, it is not clear how often these autoantibodies correlate with clinical sequelae specific to the connective tissue disease, rather than Fabry’s, as they appear to in both our described cases. The high rate of prior misdiagnosis as rheumatological disease, may in reality reflect a high rate of co-morbidity with connective tissue disease. The abnormally deposited lipid products may act as an antigen to trigger the development of autoimmunity. Autoimmune diseases must therefore always be considered when reviewing patients with Fabry disease. Both patients also highlight the difficulty of distinguishing Fabry pathology from autoimmune disease on biopsy. Myocyte enlargement due to perinuclear vacuolation and sarcoplasmic myelinoid bodies can be seen in ventricular biopsies of both Fabry disease or chloroquine toxicity. The presence of curvilinear bodies may help to distinguish cases of chloroquine toxicity. Fabry disease was diagnosed quickly in patient B because of the characteristic findings on kidney biopsy, however, patient A presented with Fabry disease affecting the heart which is less easily accessible to biopsy and subsequently her diagnosis was delayed by five years.

**Key Learning Points:** Fabry disease and autoimmune rheumatic disease can co-exist and auto-antibodies are commonly found. Symptoms usually start in adolescence with intermittent acroparasthesiae, angiokeratomas, corneal opacity, hypohidrosis, heat, cold and exercise intolerance, proteinuria, and gastrointestinal disturbance.

End-stage renal failure, severe cardiac or cerebrovascular disease are late manifestations of disease. Fabry disease is X-linked meaning females often present later with a milder phenotype. Enzyme replacement therapy is available but relies on early diagnosis.

**Disclosure: J. Hannah:** None. **S. Sangle:** None. **D. D'Cruz:** None.

